# Association between dietary phylloquinone intake and peripheral metabolic risk markers related to insulin resistance and diabetes in elderly subjects at high cardiovascular risk

**DOI:** 10.1186/1475-2840-12-7

**Published:** 2013-01-08

**Authors:** Martí Juanola-Falgarona, Jordi Salas-Salvadó, Ramon Estruch, Maria P Portillo, Rosa Casas, Jonatan Miranda, Miguel A Martínez-González, Mònica Bulló

**Affiliations:** 1 Human Nutrition Unit, Faculty of Medicine and Health Sciences, IISPV, Universitat Rovira i Virgili, C/Sant Llorenç 21, 43201, Reus, Spain; 2 CIBERobn Physiopathology of Obesity and Nutrition, Institute of Health Carlos III (ISCIII), Madrid, Spain; 3 PREDIMED Network (RD 06/0045), ISCIII, Madrid, Spain; 4 Department of Internal Medicine, Institut d’Investigacions Biomèdiques August Pi Sunyer (IDIBAPS), Hospital Clínic, Barcelona, Spain; 5 Department of Nutrition and Food Science, University of Pais Vasco, Vitoria, Spain; 6 Department of Preventive Medicine and Public Health, University of Navarra, Pamplona, Spain

**Keywords:** Vitamin K, Inflammation, Insulin resistance, Diabetes

## Abstract

**Background:**

Vitamin K has been related to glucose metabolism, insulin sensitivity and diabetes. Because inflammation underlies all these metabolic conditions, it is plausible that the potential role of vitamin K in glucose metabolism occurs through the modulation of cytokines and related molecules. The purpose of the study was to assess the associations between dietary intake of vitamin K and peripheral adipokines and other metabolic risk markers related to insulin resistance and type 2 diabetes mellitus.

**Methods:**

Cross-sectional and longitudinal assessments of these associations in 510 elderly participants recruited in the PREDIMED centers of Reus and Barcelona (Spain). We determined 1-year changes in dietary phylloquinone intake estimated by food frequency questionnaires, serum inflammatory cytokines and other metabolic risk markers.

**Results:**

In the cross-sectional analysis at baseline no significant associations were found between dietary phylloquinone intake and the rest of metabolic risk markers evaluated, with exception of a negative association with plasminogen activator inhibitor-1. After 1-year of follow-up, subjects in the upper tertile of changes in dietary phylloquinone intake showed a greater reduction in ghrelin (−15.0%), glucose-dependent insulinotropic peptide (−12.9%), glucagon-like peptide-1 (−17.6%), IL-6 (−27.9%), leptin (−10.3%), TNF (−26.9%) and visfatin (−24.9%) plasma concentrations than those in the lowest tertile (all p<0.05).

**Conclusion:**

These results show that dietary phylloquinone intake is associated with an improvement of cytokines and other markers related to insulin resistance and diabetes, thus extending the potential protection by dietary phylloquinone on chronic inflammatory diseases.

**Trial registration:**

http://www.controlled-trials.com as ISRCTN35739639

## Introduction

Vitamin K (K_1_ or phylloquinone and K2 or menaquinones) is recognized as an essential element in the synthesis of carboxylate clotting factors involved in prothrombotic disorders and cardiovascular disease. More recently, it has been reported that vitamin K also participates in the gamma-carboxylation reactions of other proteins such as osteocalcin, and may also exert a protective role against age-related bone loss [1,2]. However, additional roles of vitamin K, independent of these effects have been described [3]. Thus, there is evidence that both osteocalcin and vitamin K may have a potential beneficial role in glucose metabolism, insulin sensitivity and type 2 diabetes (T2DMs) [4-7]. Since inflammation underlies all these chronic metabolic conditions, it is plausible that the potential role of vitamin K in glucose metabolism partly occurs through the modulation of cytokines and other metabolic risk markers related to insulin resistance and diabetes.

In-vitro studies have shown an anti-inflammatory effect of vitamin K. Human macrophage THP-1 cells incubated with vitamin K reduced the interleukin-6 (IL-6) expression compared to non-incubated cells. Likewise, rats fed with a vitamin K-deficient diet showed an enhanced expression of genes involved in the acute inflammatory response [8]. In a subsample of 1,321 subjects from the Framingham Offspring Study, both plasma phylloquinone and dietary phylloquinone intake were inversely associated with peripheral concentrations of some inflammatory markers [9]. However, in a 3-year randomized clinical trial designed to assess the effect of vitamin K supplementation on bone loss, no differences were found in the plasma IL-6, C-reactive protein or osteoprotegerin concentrations of participants receiving or not a phylloquinone supplement [10].

The purposes of the present study were to assess the cross-sectional associations between dietary intake of vitamin K1 and selected adipokines or other metabolic risk markers related to inflammation, insulin resistance and diabetes; and to longitudinally analyse the associations between changes in dietary phylloquinone intake and changes in these risk markers after one-year of follow-up in a cohort of elderly subjects at high cardiovascular risk.

## Methods

### Study population

In the present study we conducted a cross-sectional and a longitudinal assessment of 568 consecutively recruited participants for the PREDIMED trial centers of Reus and Barcelona (Spain). The PREDIMED study is a large, parallel group, multicenter, controlled, randomized, clinical trial designed to evaluate the effect of the Mediterranean diet on the primary prevention of cardiovascular disease in elderly. Participants were community-dwelling men and women aged 55–80 and 60–80 years, respectively. At baseline they were free of cardiovascular disease and were either diabetic or met at least three or more coro-nary heart disease risk factors including smoking, hypertension (blood pressure ≥ 140/90 mmHg or treatment with antihypertensive drugs), dyslipidemia [low-density lipoprotein cholesterol level ≥ 160 mg/dL or treatment with hypolipidemic drugs], high-density lipoprotein cholesterol level of 40 mg/dL or lower, overweight [Body mass index ≥ 25 kg/m^2^ or family history of premature cardiovascular disease. Exclusion criteria included any severe chronic illness, drug or alcohol addiction, history of allergy or intolerance to olive oil or nuts, or a low predicted likelihood of changing dietary habits according to Prochaska and DiClemente’s stages-of-change model. The participants included in the PREDIMED study were randomly assigned to 3 intervention groups: a Mediterranean Diet with virgin olive oil, a Mediterranean Diet with mixed nuts and a control group where a low-fat diet is recommended according to the American Heart Association guidelines. Full details of the PREDIMED study protocol have been published elsewhere [11,12]. The study protocol was approved by the institutional review boards of Hospital Clínic and Hospital Universitari Sant Joan de Reus, and all subjects agreed to participate in the study and gave their written informed consent. The trail was registered in http://www.controlled-trials.com as ISRCTN35739639.

### Dietary assessment

Two individual motivational interviews every 3 months to negotiate nutrition goals, and group educational sessions on a quarterly basis, focused to adapt the customary diet to a traditional Mediterranean diet, were compared with a control group, which received verbal instructions and a leaflet recommending the National Cholesterol Education Program Adult Treatment Panel III dietary guidelines (http://www.predimed.org). At baseline and after one-year of follow-up participants were assessed by trained dieticians who administered a previously validated 137-item food frequency questionnaire (FFQ) [13]. Additionally, a validated brief 14-item Mediterranean Diet Adherence Screener was used to assess adherence to the traditional Mediterranean Diet (MedDiet) where subjects were asked for their consumption of the most common Mediterranean foods [14]. Subjects with a higher consumption of healthier foods such as olive oil, vegetables, legumes, fruit, nuts, fish and seafood, white meat instead of red meat, *sofrito* and red wine scored higher in this questionnaire. Energy and nutrient intakes were calculated from Spanish food composition tables [15,16]. Dietary phylloquinone intake was calculated using the database of the US Department of Agriculture, Human Nutrition Research Center on Aging at Tufts University (http://www.nal.usda.gov/fnic/foodcomp/search) and the reproducibility and relative validity of a self-administered FFQ used in the study was validated for dietary phylloquinone intake. Reproducibility for dietary phylloquinone intake explored by the Pearson correlation coefficient (r) ranged was 0.755, and the intraclass correlation coefficient (ICC) was 0.860, p<0.001.

### Other measurements

Additional information was collected on subjects’ medical record, including the use of medication. Trained personnel measured baseline weight, height and waist circumference as previously reported [11,12], as well as blood pressure in triplicate with a validated semiautomatic oscillometer (Omron HEM-705CP, Hoofddorp, the Netherlands). Leisure-time physical activity was evaluated using the validated Spanish version of the Minnesota leisure-time physical activity questionnaire. Centralized laboratory biochemical analyses were performed on blood samples obtained in fasting conditions. Plasma glucose, serum cholesterol, high-density lipoprotein cholesterol and triglyceride concentrations were determined using standard enzymatic automated methods. In patients whose triglyceride levels were less than 400 mg/dL, low-density lipoprotein cholesterol concentrations were estimated using the Friedewald formula. Inflammatory and metabolic markers (adiponectin, adipsin, C-peptide, ghrelin, glucagon-like peptide-1 (GLP-1), glucose-dependent insulinotropic polypeptide, IL-6, leptin, plasminogen activator inhibitor-1 (PAI-1), resistin, tumor necrosis factor (TNF) and visfatin were determined in plasma using the Bio-Plex cytokine assay (Bio-Rad Laboratories Inc., Hercules, CA, USA) according to manufacturer’s instructions.

### Statistical analysis

Mean (SD) or percentages (%) were used to describe the participant’s baseline characteristics. Inflammatory and metabolic risk markers of insulin resistance and diabetes were logarithmically transformed to achieve a normal distribution, and the geometric mean and 95% confidence interval were used to describe these variables. For cross-sectional associations, we used multivariable linear regression models to assess the associations between metabolic risk markers (dependent variables in each model) and dietary vitamin K intake (independent variable in all models) adjusted for potential confounding variables [age, sex, body mass index, smoking (never, current, past), physical activity (kcal/d), type 2 diabetes mellitus (T2DM), total energy (kcal/d) and fibre intake (g/d), dietary polyunsaturated fatty acids (PUFA) intake (g/d) and adherence to MedDiet (14-item score, quantitative)]. The selection of potential confounders was done using clinical plausible and bibliographical criteria.

Interaction tests for sex and T2DM (sex*vitamin K intake, T2DM* vitamin K intake) were not statistically significant. No interaction was observed for intervention group and changes in dietary phylloquinone intake in any outcome (inclusion of intervention group*one-year changes in dietary phylloquinone intake in the regression models). In the longitudinal analyses, subjects were categorized according to tertiles of changes in dietary vitamin k intake from baseline to 1-year follow-up. A multivariable linear regression model was fitted to evaluate the relationship between metabolic risk markers at 1-year of follow-up (dependent variables in each model) and tertiles of change in vitamin k intake (independent variable in all models) adjusting by age, sex, smoking (never, current, past), physical activity (kcal/d), T2DM, intervention group, baseline values of each metabolic marker and changes in BMI, total energy intake (kcal/d), fiber intake (g/d), dietary PUFA intake (g/d) and adherence to MedDiet (the relative change in the 14-item score was expressed as a percentage). All statistical tests were two-tailed, and the significance level was p<0.05. Statistical analysis was performed using SPSS 17.0 for Windows (SPSS Inc, Chicago, IL).

## Results

Of the 568 subjects consecutively recruited, 57 were excluded because they were using anti-inflammatory medication at baseline and 1 because he hd not completed the FFQ at follow-up. Table 1 summarizes the baseline characteristics of the study participants. Study subjects were 67.2±6.0 years old and 44.4% of them were male. Most of them were overweight or obese (92.2%), had hypertension (91.8%), were hypercholesterolemic (62.9%), and 55% had T2DM. Table 2 presents the baseline and 1-year dietary changes by tertiles of change in dietary phylloquinone intake. Subjects in the highest tertile of change consumed less amounts of vitamin K1 at baseline. This change after intervention was due to a higher consumption of total vegetables and, especially, leafy green vegetables, the primary dietary source of vitamin K1. In cross-sectional analyses at baseline, a negative significant association was found between dietary phylloquinone intake and PAI-1 plasma concentrations, even after adjusting for potential confounders (Table 3), but not for the rest of the metabolic risk markers. However, after 1-year of follow-up, those subjects in the upper tertile of changes in dietary phylloquinone intake showed a significant greater reduction in ghrelin (15.0%), GIP (12.9%), GLP-1 (17.6%), IL-6 (27.9%), leptin (10.3%), TNF (26.9%) and visfatin (24.9%) than those subjects in the lowest tertile (Table 4). No significant associations were found between changes in dietary phylloquinone intake and other metabolic markers of inflammation, insulin resistance and diabetes.

**Table 1:** Baseline characteristics of study subjects by tertiles of change in dietary phylloquinone intake (μg/day)

**Characteristics**	**All subjects (n=510)**			**P**
	**Tertile 1**	**Tertile 2**	**Tertile 3**	
	**(−718.2 to −69.9)**	**(−26.8 to 69.4)**	**(70.5 to 767.5)**	
**Clinical characteristics**				
**Men/women, n**	80/92	68/107	78/85	0.194
**Age, years**	66.8 ± 6.1	68.1 ± 6.2	66.6 ± 5.7	0.030
**BMI, kg/m**^**2**^	29.39 ± 3.17	29.21 ± 3.18	29.17 ± 3.05	0.608
**Waist circumference, cm**	100.6 ± 8.6	100.5 ± 9.2	100.9 ± 8.9	0.940
**Current smoker, n (%)**	26 (15.1)	29 (16.6)	16 (9.8)	0.561
**Type 2 diabetes, n (%),**	95 (55.2)	100 (57.1)	85 (52.1)	0.650
**Overweight/Obesity, n (%)**	159 (92.4)	162 (92.6)	149 (91.4)	0.911
**Hypertension, n (%)**	156 (90.7)	164 (93.7)	148 (90.8)	0.121
**Dyslipydemia, n (%)**	103 (61.7)	116 (68.6)	92 (57.9)	0.511
**Intervention group MD+VOO/MD+nuts/CD, n**	44/54/74	64/57/54	76/45/42	0.001
**Leisure-time physical activity, METS-min/day**	277.8 ± 261.9	263.0 ± 247.8	294.2 ± 279.4	0.313
**Metabolic risk markers**				
**C-Peptide (ng/mL)**	1.41 (1.34 to 1.49)	1.33 (1.24 to 1.44)	1.34 (1.25 to 1.42)	0.374
**Ghrelin (pg/mL)**	12.88 (11.95 to 3.89)	13.38 (12.31 to 14.54)	12.77 (11.75 to 13.87)	0.685
**GIP (pg/mL)**	92.55 (84.76 to 101.05)	98.40 (89.70 to 107.95)	85.72 (78.25 to 93.91)	0.107
**GLP-1 (ng/mL)**	1.22 (1.09 to 1.36)	1.31 (1.17 to 1.48)	1.16 (1.03 to 1.30)	0.312
**IL-6 (pg/ml)**	9.96 (8.80 to 11.27)	10.88 (9.63 to 12.29)	8.99 (7.93 to 10.19)	0.102
**Leptin (ng/mL)**	2.87 (2.60 to 3.17)	3.20 (2.88 to 3.57)	2.91 (2.63 to 3.22)	0.262
**PAI-1 (ng/mL)**	3.20 (3.05 to 3.37)	3.28 (3.11 to 3.46)	3.37 (3.21 to 3.54)	0.378
**Resistin (ng/mL)**	0.98 (0.92 to 1.05)	1.05 (0.98 to 1.12)	1.01 (0.94 to 1.08)	0.413
**TNF (pg/mL)**	13.36 (11.57 to 15.44)	13.76 (11.89 to 15.93)	11.35 (9.80 to 13.14)	0.145
**Visfatin (ng/mL)**	4.13 (3.53 to 4.84)	4.54 (3.85 to 5.37)	3.70 (3.13 to 4.37)	0.225
**Adiponectin (μg/mL)**	48.87 (42.67 to 55.98)	49.51 (42.68 to 57.43)	42.37 (35.45 to 50.64)	0.302
**Adipsin (μg/mL)**	1.12 (1.01 to 1.24)	1.14 (1.03 to 1.28)	0.91 (0.77 to 1.08)	0.029

Data are given as mean (SD) or number (%) unless otherwise indicated. Metabolic risk markers are expressed as geometric means (IC95%). P values of the difference between tertils of change in dietary phylloquinone intake (ANOVA for the continuous variables and a *χ*2 test for categorical variables). *MD+VOO* Mediterranean diet + Virgin Olive Oil, *MD+nuts* Mediterranean diet + Nuts, *CD* Control diet, *BMI* body mass index, *GLP-1* glucagon-like peptide 1, *GIP* glucose-dependent insulinotropic polypeptide; *IL-6* interleukin-6, *PAI-1* plasminogen activator inhibitor-1, *TNF-α* tumor necrosis factor-α.

**Table 2:** Baseline and 1-year change of dietary characteristics by tertiles of change in dietary phylloquinone intake (μg/day)

	**Tertile 1**	**Tertile 2**	**Tertile 3**	**P**
	**(n=172)**	**(n=175)**	**(n=163)**	
**Total energy intake, (kcal/d)**				
Baseline	2526 ± 557	2359 ± 547	2270 ± 514	<0.001
Change	−85 ± 561	86 ± 30	198 ± 521	<0.001
**Energy from total protein, (% kcal)**				
Baseline	17 ± 3	17 ± 3	17 ± 3	0.386
Change	0 ± 3	−1 ± 3	0 ± 3	0.134
**Energy from total carbohidrates, (% kcal)**				
Baseline	42 ± 6	42 ± 7	42 ± 7	0.982
Change	−2 ± 6	−2 ± 7	−2 ± 8	0.958
**Fiber intake, g/1,000 kcal**				
Baseline	12 ± 4	12 ± 3	11 ± 3	0.023
Change	−1 ± 3	0 ± 2	2 ± 3	<0.001
**Energy from total fat, (% kcal)**				
Baseline	39 ± 6	39 ± 6	39 ± 6	0.393
Change	2 ± 6	3 ± 7	2 ± 8	0.240
**Saturated fatty acids, (%)**				
Baseline	27 ± 4	26 ± 5	26 ± 4	0.829
Change	−2 ± 5	−3 ± 5	−2 ± 5	0.271
**MUFA, (%)**				
Baseline	49 ± 5	48 ± 5	49 ± 5	0.242
Change	1 ± 6	2 ± 5	1 ± 6	0.140
**PUFA, (%)**				
Baseline	17 ± 4	17 ± 4	16 ± 4	0.456
Change	0 ± 5	1 ± 5	1 ± 5	0.221
**Phylloquinone intake, (μg/d)**				
Baseline	490 ± 229	401 ± 209	297 ± 163	<0.001
Change	−176 ± 150	16 ± 25	266 ± 164	<0.001
**Vegetable consumption, (g/d)**				
Baseline	413 ± 167	380 ± 182	335 ± 186	<0.001
Change	−42 ± 180	17 ± 131	141 ± 175	<0.001
**Leafy green vegetables, (g/d)**				
Baseline	112 ± 47	95 ± 47	75 ± 44	<0.001
Change	−30 ± 38	1 ± 20	55 ± 44	<0.001
**Other vegetables, (g/d)**				
Baseline	276 ± 138	265 ± 142	239 ± 147	0.053
Change	−13 ± 149	7 ± 120	79 ± 153	<0.001
**Fruit consumption, (g/d)**				
Baseline	479 ± 258	451 ± 240	432 ± 251	0.221
Change	−12 ± 223	36 ± 211	67 ± 238	0.005
**Legume consumption, (g/d)**				
Baseline	20 ± 11	19 ± 12	17 ± 9	0.046
Change	1 ± 12	4 ± 12	7 ± 13	<0.001
**Cereal consumption, (g/d)**				
Baseline	270 ± 103	256 ± 102	249 ± 103	0.150
Change	−27 ± 100	−10 ± 119	−1 ± 113	0.091
**Dairy product consumption, (g/d)**				
Baseline	357 ± 227	371 ± 252	349 ± 195	0.656
Change	−8 ± 212	−8 ± 190	2 ± 181	0.854
**Meat consumption, (g/d)**				
Baseline	154 ± 61	148 ± 61	143 ± 56	0.243
Change	−14 ± 58	−12 ± 62	−2 ± 61	0.137
**Fish consumption, (g/d)**				
Baseline	115 ± 46	106 ± 46	106 ± 42	0.099
Change	−1 ± 52	9 ± 46	15 ± 43	0.008
**Alcohol intake, (g/d)**				
Baseline	13 ± 19	10 ± 19	7 ± 12	0.013
Change	0 ± 13	−1 ± 17	1 ± 9	0.173
**Olive oil consumption, (g/d)**				
Baseline	39 ± 15	36 ± 14	38 ± 15	0.248
Change	5 ± 19	11 ± 19	9 ± 20	0.021
**Nut consumption, (g/d)**				
Baseline	16 ± 17	14 ± 15	11 ± 13	0.029
Change	7 ± 25	10 ± 25	11 ± 24	0.185
**14-item PREDIMED MedDiet Score**				
Baseline	9 ± 2	9 ± 2	8 ± 2	0.443
Change	1 ± 2	1 ± 2	2 ± 2	0.031

Data expressed as mean ± standard deviation. ANOVA was used for analysis of the difference between tertils of change in vitamin K intake. Abbreviations: *MUFA* monounsaturated fatty acids, *PUFA* polyunsaturated fatty acids, *MedDiet* Mediterranean diet.

**Table 3:** Cross-sectional associations between intake of 100 μg of dietary phylloquinone and inflammatory or metabolic markers at baseline

	**Change in inflammatory marker for 100 additional μg of dietary phylloquinone intake (95%confidence intervals)**	**P**
**C-Peptide (ng/mL)**	−0.73 (−2.68 to 1.27)	0.472
**Ghrelin (pg/mL)**	−1.25 (−3.76 to 1.33)	0.339
**GIP (pg/mL)**	−0.13 (−2.98 to 2.80)	0.928
**GLP-1 (ng/mL)**	−1.81 (−5.40 to 1.91)	0.335
**IL-6 (pg/ml)**	−1.68 (−5.46 to 2.25)	0.396
**Leptin (ng/mL)**	−1.48 (−3.80 to 0.90)	0.221
**PAI-1 (ng/mL)**	−1.64 (−3.23 to −0.0)2	0.047
**Resistin (ng/mL)**	−1.42 (−3.61 to 0.81)	0.210
**TNF (pg/mL)**	−1.24 (−5.73 to 3.46)	0.598
**Visfatin (ng/mL)**	−1.42 (−6.54 to 3.98)	0.599
**Adiponectin (μg/mL)**	2.45 (−2.23 to 7.35)	0.310
**Adipsin (μg/mL)**	1.21 (−2.84 to 5.43)	0.563

Multivariable linear regression models were used for analysis. Data are given in % of change (95% IC). Models are adjusted for sex, age, body-mass-index, smoking (never, current, past), total energy intake (kcal/d), dietary fibre intake (g/d), dietary polyunsaturated fatty acids intake (g/d), physical activity (kcal/d), Mediterranean Diet Score (14-item PREDIMED score, quantitative), and T2DM. *GIP* Gastric inhibitory polypeptide, *GLP-1* Glucagon-like peptide-1, *IL-6* Interleukin-6, *PAI-1* Plasminogen activator inhibitor-1, *TNF* Tumor Necrosis Factor α.

**Table 4:** Longitudinal associations between one-year changes in inflammatory or metabolic markers and tertiles of change in dietary phylloquinone intake (μg/day)

	**Tertile 1**	**Tertile 2**	**p**	**Tertile 3**	**p**	**p for trend**
**C-Peptide (ng/mL)**	Ref.	−2.19 (−8.35 to 4.40)	0.505	−6.10 (−12.61 to 0.91)	0.086	0.083
**Ghrelin (pg/mL)**	Ref.	1.69 (−8.58 to 13.11)	0.758	−15.00 (−24.49 to −4.32)	0.007	0.003
**GIP (pg/mL)**	Ref.	0.08 (−10.17 to 11.49)	0.989	−12.94 (−22.75 to −1.89)	0.023	0.014
**GLP-1 (ng/mL)**	Ref.	0.21 (−12.99 to 15.42)	0.977	−17.64 (−29.67 to −3.56)	0.016	0.009
**IL-6 (pg/ml)**	Ref.	−4.50 (−18.17 to 11.46)	0.559	−27.89 (−39.20 to −14.48)	<0.001	<0.001
**Leptin (ng/mL)**	Ref.	−7.79 (−15.61 to 0.76)	0.073	−10.26 (−18.62 to −1.03)	0.030	0.044
**PAI-1 (ng/mL)**	Ref.	−3.06 (−10.65 to 5.17)	0.454	−3.24 (−11.61 to 5.93)	0.476	0.529
**Resistin (ng/mL)**	Ref.	−2.43 (−11.03 to 7.01)	0.601	−6.66 (−15.71 to 3.35)	0.184	0.180
**TNF (pg/mL)**	Ref.	−3.96 (−20.47 to 15.98)	0.674	−26.89 (−40.65 to −9.94)	0.003	0.002
**Visfatin (ng/mL)**	Ref.	−8.08 (−24.73 to 12.24)	0.408	−24.90 (−39.86 to −6.22)	0.012	0.010
**Adiponectin (μg/mL)**	Ref.	−3.27 (−19.20 to 15.79)	0.716	−7.34 (−23.98 to 12.93)	0.449	0.452
**Adipsin (μg/mL)**	Ref.	−2.45 (−15.37 to 12.45)	0.732	−7.39 (−20.90 to 8.44)	0.340	0.332

Multivariable linear regression models were used for analysis. Data are given in % of change (95% IC). Models are adjusted for sex, age, change of body-mass-index, smoking (never, current, past), change of total energy intake (kcal/d), change of fibre intake (g/d), change of dietary polyunsaturated fatty acids intake (g/d), physical activity (kcal/d), intervention group, change of Mediterranean Diet Score (%), T2DM and baseline values of each metabolic marker. *GIP* Gastric inhibitory polypeptide, *GLP-1* Glucagon-like peptide-1, *IL-6* Interleukin-6, *PAI-1* Plasminogen activator inhibitor-1, *TNF*, Tumor Necrosis Factor α.

## Discussion

The results of this study show, for the first time, that an increased dietary intake of phylloquinone is associated with an improvement in inflammatory and other metabolic risk markers related to insulin resistance and diabetes, thus supporting a protective role of vitamin K on low-grade chronic inflammatory diseases.

In recent years, vitamin K has been attributed a putative role in glucose metabolism, insulin resistance and T2DM [17]. Recently, in a prospective study our group has shown that a higher dietary phylloquinone intake was associated to a lower risk of developing T2DM. However, the exact mechanisms underlying this relationship remain still unknown. One potential explanatory mechanism could be related to the role of vitamin K as a cofactor in the carboxylation of vitamin K-dependent proteins, such as osteocalcin [18], GAS6 or Protein S [19] and through their role on NF-kB [20-22]. Additionally, some authors have suggested that inflammation may be modulated by a possible antioxidant effect of vitamin K [23].

A cross-sectional study conducted in a subsample from the Framingham Offspring Study, showed an inverse association between 2-fold changes in usual dietary phylloquinone intake or plasma phylloquinone concentrations and seven of the fourteen peripheral inflammatory markers measured [9]. However, the same authors, in a cross-sectional analysis conducted on 400 healthy elderly men and women, did not find any significant association between plasma phylloquinone concentrations and C-reactive protein or IL-6 after adjusting for potential confounders [10]. Similar to the results from Shea and co-workers, we also failed to find any significant association between dietary phylloquinone intake and most of the metabolic risk markers analysed. However, our longitudinal findings are generally consistent with in-vitro studies or those of a previous epidemiological study. In-vitro studies found that the production of the proinflammatory cytokines IL-6 or TNF by human gingival fibroblast or mouse macrophage cells, respectively, decreased when cells were incubated with different vitamin K family compounds [21,24]. Other studies have found that vitamin K suppresses inflammation by lowering the expression of genes for some proinflammatory cytokines, such as IL-6, IL-1β and TNF [8,22]. However, only a single 3-year, double-blind, randomized controlled trial has been conducted to evaluate the effect of vitamin K supplementation on peripheral inflammatory marker concentrations. In that study, conducted with 379 healthy men and women, no significant relationship between plasma phylloquinone levels and inflammation markers was shown [10]. In our study we observed a significant improvement in the inflammatory status (leptin, IL-6, TNF) and a decrease in other metabolic risk markers related to insulin resistance and diabetes such as visfatin, ghrelin, GIP and GLP-1 among subjects who increased their dietary intake of phylloquinone after 1-year follow-up, thus contributing to extend the knowledge on the role of vitamin K in humans and to support our previous results on dietary phylloquinone intake and T2DM incidence [7]. The discrepancies between our results and those previously published could be partly explained by the differences between the populations. Our participants were elderly subjects at high cardiovascular risk, whereas, subjects in the study of Shea [9] were healthy and generally free of chronic disease. This may contribute to explain why inflammatory cytokines remained unchanged in that study. Moreover, in our study we assessed the association of inflammation with dietary phylloquinone intake instead of plasma phylloquinone as was done in the previous study. Whether circulating levels of phylloquinone are correlated to dietary phylloquinone intake remains to be elucidated.

It must be noted that leptin, IL6 and TNF are pro-inflammatory cytokines with a recognised role in the development and progression of insulin resistance, T2DM and cardiovascular disease [25,26]. Also visfatin and ghrelin appears as important mediators of inflammation in addition to glucose-lowering and insulin-mimicking/sensitizing effects or a suppressive role of ghrelin in the release of insulin from the pancreatic islets [27-30]. A strong inverse correlation between plasma ghrelin concentrations and insulin resistance has been observed in several studies [30-32] and lower concentrations of ghrelin have been observed in T2DM subjects [33]. The negative relationship between changes in dietary phylloquinone intake and plasma incretin concentrations observed in our study could be explained because a higher intake of phylloquinone may promote better glycemic control thus leading less necessary the glucose and insulin regulation mediated by incretins. However, the pleiotropic role recently attributed to incretins could also contribute to explain our results. Although there is growing evidence that incretin hormones (GIP and GLP-1) simulate glucose-dependent insulin secretion and stimulate pancreatic synthesis of insulin, a novel link between inflammation and incretin hormones has been proposed. First, IL-6 increase GLP-1 production in intestinal L cells and alpha pancreatic cells improving insulin secretion and glycemia [34]. Therefore, the reduction of IL-6 levels observed in the subjects allocated in the highest tertile of change in phylloquinone intake could partly explain the reduction in GLP-1 concentrations in this group. Nie et al., have also demonstrated the capacity of GIP to activate inflammatory response and promote secretion of pro-inflammatory cytokines and chemokines in cell culture adipocytes [35]. Additionally, a potential role of GIP on adipose tissue insulin resistance mediated by osteopontin regulation has also been suggested [36]. The results from our study could be related to the potential role of incretins on adipose tissue in a fasting situation rather than to their established role on pancreatic cells in a post-prandial state. Whether or not GIP and GLP-1 need to be considered as new adipokynes or related pro-inflammatory markers could not be elucitaded from the results of our study. Specific studies are needed to get a deeper understanding of the exact role of incretins on adipose tissue and their interaction with the rest of adipokynes.

Our study has several limitations. It should be kept in mind that the subjects in our study were randomly allocated to a healthy Mediterranean diet that could partially account for the reduction in peripheral metabolic risk markers in some of them although no significant interaction between intervention group and dietary phylloquinone intake was observed for any of the outcomes. In order to minimize the potential effect of a healthy dietary pattern on inflammatory response we have adjusted the regression models for the adherence to a MedDiet. Subjects in the PREDIMED study reported a higher dietary intake of phylloquinone than in other epidemiological studies, probably because this study was conducted in a Mediterranean country where the consumption of fruit and vegetables is high. In populations with a lower consumption of phylloquinone or poor nutrition an increase in dietary vitamin K1 would probably be much more beneficial. The cohort studied was elderly and at high risk of cardiovascular disease, so our findings cannot be generalized to younger or healthier individuals. Because there is no perfect correlation between dietary phylloquinone intake and its absorption it would be interesting in the future to evaluate the associations showed in our study not only with dietary phylloquinone intake but also with a circulating marker of vitamin K status. Finally, we cannot discount a slight overestimation of dietary phylloquinone intake due to the use of FFQ and the USDA Food Database Composition. Although the FFQ used in our study was not specifically validated for phylloquinone intake, the intraclass correlation coefficient of vegetables (the main source of dietary vitamin K1) was 0.81, one of the highest coefficients obtained during the validation of the FFQ in the PREDIMED cohort [13]. Balanced against these limitations, the main strength of our study is its longitudinal design, which enables us to suggest a cause-effect relationship between changes in dietary phylloquinone intake and changes in inflammatory and related metabolic risk markers. In addition, our study was conducted in a large sample of individuals, and measured a panel of adipokines and related molecules involved in inflammation, glucose metabolism and cardiovascular risk.

In summary, our results support that an increase in dietary phylloquinone intake can lead to an improvement in inflammation and inflammatory-related molecules and also support the contention that high vitamin K1 intake has a beneficial effect on cardiovascular disease and other inflammation-related disorders.

## Abbreviations

FFQ: Food Frequency Questionnaire; GLP-1: Glucagon-like peptide 1; IL: Interleukin; MedDiet: Mediterranean Diet; PAI-1: Plasminogen activator inhibitor-1; T2DM: Type 2 diabetes mellitus; TNF: Tumor necrosis factor alpha.

## Competing interests

The authors declare that they have no competing interests.

## Authors’ contributions

JS, RE, MAM and MB contributed to the study design, study performance, data analysis and writing of the manuscript; MJ-F and RC performed biochemical measurements and contributed to the data analysis and the writing of the manuscript. MP and JM revised the manuscript critically for important intellectual content. All authors had a substantial input in critically appraising the manuscript and approved the final version to be published. All authors read and approved the final manuscript.
